# The Endoscope, and Its Application to the Diagnosis and Treatment of Urinary Affections

**Published:** 1867

**Authors:** A. J. Desormeaux

**Affiliations:** Surgeon of the Hospital, Member of the Surgical and Anatomical Societies of Paris


### THE ENDOSCOPE,

AND ITS APPLICATION TO THE DIAGNOSIS AND TREATMENT OF

### URINARY AFFECTIONS.

LESSONS GIVEN AT NECKER HOSPITAL.

BY

A. J. DESORMEAUX,

SURGEON OF THE HOSPITAL, MEMBER OF THE SURGICAL AND ANATOMICAL SOCIETIES OF PARIS.

TRANSLATED BY

R. P. HUNT, M.D.

### DEDICATION.

To Mons. Rayer:—

*Much Honored Master:*—According to your advice, I have given these lessons. You thought they might be useful, and encouraged their publication. Permit me here, to place them under your protection and thank you, in advance, for the aid your name will give them.

A. J. DESORMEAUX.

### TRANSLATOR'S DEDICATION.

Monsieur Rayer—To you, from whom I have learned so much of the little that I do know, permit me, as a mark of gratitude, to place your name at the head of this paragraph. R. P. HUNT.

### PREFACE.

Nos quoque oculos eruditos habemus.—*Cic. Parad.* v.

A most characteristic progression of the medical art, as it exists at present, consists in the endeavor to find out means adapted to the direct exploration of the different organs, the careful study of local affections by the aid of these means, and the use we can make of the physical sciences, from which we ask no more empty theories, but the application of their exactness and the means by which to procure instruments calculated to increase and perfect the action of our organs. Percussion, instituted by Anembrugger, popularized by Corvisart, seems to be the initiative of this path into medicine. We all know to what extent this method has reached at present; but yet it was only in its young infancy when auscultation was discovered and comes, scarcely yet invented, to revolutionize the curing art. Lænnec, a master of sciences, without counting this invention, which has immortalized his name, has had the glory, possibly unique, of creating, unaided, an entire branch of the art, which numerous and learned physicians, by constant study and effort, have only been able to extend and perfect since his time.

At the same time, Recamier, taking up the *speculum*, at that time unused, made from it his invention, rather, from the information it afforded him than from giving it a simpler and more convenient form. Collaborators were not wanting to complete his work:—First, I will mention my father, Professor Desormeaux, who found in this instrument a means of still further extending his knowledge, already great, in this branch of medicine. Later, M. Ricord, applying the speculum to the necessities of his specialty, used it for the purpose of establishing a fundamental point of doctrine. In a memoir read to the Academy of Medicine, he showed the possibility of discovering, even within the uterine neck, the characteristic ulceration of syphilis, which he distinctly separates from menorrhagia. At the same time, Mr. Milier modified the instrument, so as to adopt it to the various indications which it can well fulfil, used it for the purpose of enriching, by new means, therapeutics, as applied to affections of the tissues.

It was much, doubtless, to subject the interior organs to a free access of light, but still it was not sufficient; there are some parts so arranged that they cannot be examined in so simple a mode; the eye can only reach them, so to speak, by forcing its light upon them, and light borrowed from the science of physics. For the eye, the *ophthalmoscope* has been invented, *laryngoscopes* for the study of the upper part of the aëriferous tubes, and, for those cavities too narrow to admit the introduction of a speculum, I propose the *endoscope.* The first I drew from it, was separating menorrhagia from other urethral discharges, and if M. Ricord should see these lessons, he will recognize, I hope, that the *endoscope* has furnished what he desired, in his letters, from the urethroscope, to a former pupil.

When, in 1862, I was made surgeon to Necker Hospital, I found many occasions on which to continue my researches, and the *endoscope*, up to that time an experiment, soon became an instrument of practical importance, as relates to diagnosis and treatment, and was, the following year, the subject of several articles in the medical journals.

I owe, here, a special mention of a thesis made this year, by my friend and former pupil, Dr. Portella (de Fernambouc), upon *endoscopic urethrotomy*. This is the first publication on the new method of incision for stricture.

J. A. DESORMEAUX.

---

### THE ENDOSCOPE.

### FIRST LECTURE.

*The Endoscope.—The Healthy Urethra.—Acute Urethritis.*

GENTLEMEN:—When we consider all the improvements and inventions, having in view diseases of the urinary organs, it may be supposed that this is one of the most advanced parts of surgery; but a judicious examination of the subject proves the reverse.

Really, despite the improvements added to this branch of our art, despite the numerous researches so long continued, there are still many obscurities, many diagnostic uncertainties, and much risk in the treatment. Of this, the following pages will furnish abundant proof. But, at present, let me call your attention to calculi sacked (*enchatonnæ*), the frequence of which is so often disputed; some believe these cases very rare, others are of an opposite opinion. One, of long and extensive practice tells me that they do not exist, and another, of great special experience, regards them as very rare. If this is so, I have been most singularly favored, for twice I have found them in operating, and twice, in the last year, the endoscope has shown them; once upon a patient of Mr. Howel, at the clinic, and once upon a patient of M. Jarjavay, at St. Antoine. M. Howel proved his case by an autopsy, M. Jarjavay verified his by an an operation. Now, is it probable, if these cases are reported as so rare, that it is owing to imperfect means of discovering them?

Let us take, as another example, a very frequent disease. Nothing is more common than chronic urethral discharge. In general language, blenorrhœa, the dread of surgeons, and of patients until they determine to forget their disease and live tranquilly, thoughtless of such accidents as may one day awake them from their security; for if there are cases of blenorrhœa which spontaneously cure, or which disappear to soon return without producing much damage, there are others, and many, which end only by producing most of the grave affections of the urinary organs.

Why have we not distinguished between these different kinds of blenorrhœa?

When a surgeon is consulted for an eye, red, painful, weeping, he seeks to discover the nature of the disease, he desires to know if he is to treat an ophthalmia blenorrhagic, syphilitic, catarrhal, rheumatismal, scrofulous, or simply traumatic; he is never content to diagnose simply an ophthalmia, and still less a hyper-secretion of the conjunctiva. In chronic discharges from the vagina, leucorrhœa furnishes a very insufficient term for diagnosis.

Why then should we be less difficult to be satisfied as regards urethral discharges, and apply to them, indiscriminately, the term of blenorrhœa, or confound them with catarrh, as if it was not an affection *sui generis*, in the urethra as in other organs, rarer only on the urethral than other mucous surfaces? It is, because in ophthalmia, by opening the lids to see the diseased organ, we see and conjoin the physical and rational signs. In vaginal discharge, the speculum and actual touch combined, is sufficient. In the urethra, however, sight has not, until recently, aided us to complete a diagnosis firmly based. We stand where the profession stood, on the question of leucorrhœa, before Recamier used the speculum in the study of affections of the vagina and uterus, with the difference that in place of a finely exquisite finger, touch can only be applied by means of inert instruments.

We spoke, a few moments since, of the different ophthalmias, to show what difference exists in the pathology of organs as they may be more or less accessible to view. Deep-seated affections of the eye will furnish a still better illustration. A few years since, every patient devoid of sight, without opacity of the pupil, was pronounced to have amaurosis if the pupil was black, glaucoma if the pupil had a greenish tint. The diagnosis was not difficult, but what is glaucoma, and what amaurosis? This was not known. As to glaucoma, there was complete ignorance. As to amaurosis, we know that the term covered many different affections, some seated in the eye, others elsewhere. Pathological anatomy demonstrated some of them, but they could not be distinguished on the living subject, and a haphazard, empirical treatment was resorted to. Until Helmtroty invented the ophthalmoscope, this uncertainty continued. This instrument was used for deep-seated affections of the eyes which, up to that date, could only be diagnosed by rational signs—the result is well known.

Glaucoma is at present considered a well-determined and well-decided lesion, easily understood by its symptoms, and of which a rational treatment has been instituted with some success. Under that term, many different affections have been discovered, which, studied separately, have much narrowed the domain of amaurosis, and will still more restrict it. Ask a countryman of Helmtrothy, to-day, what is amaurosis, and he will not tell you "that it is a disease of the eyes, in which the surgeon sees no more than the patient."

At the same time that the ophthalmoscope was commencing to be employed in Germany, a lucky circumstance, unnecessary to relate here, caused me too seek for an instrument that would permit of ocular examination of the urethra and bladder. The principle was soon found, but months passed before the URETHROSCOPE became the ENDOSCOPE. I ought to state that I was not the first to make attempts on this road. At the time the speculum commenced its revolution in uterine diseases, M. Sigelas attempted to obtain, for the bladder, similar advantages, by means ef two concentric sounds, the inner one to permit light to reach to the depth of the viscous, and the exterior to transmit the light of two candles reflected from a concave mirror. The results were not very satisfaatory; yet, the inventor, by modifying his instrument, might have gained his end, if other work had not taken his time and attention. I learn from M. Ségalas, that when he presented to the Academy of Sciences his urethro-cystic speculum, Fresnel, on leaving, said to him, by placing the light at the side, and, by means of an inclined mirror, reflecting it according to the axis of the instrument, you will succeed. Such is the arrangement in the endoscope.

So far as I can learn, an English surgeon, M. Every, imagined an instrument mnch like mine, but it was never used. Of this instrument, I have never been able to procure a detailed account.

Then, Dr. Ag. Hacken, of Riga, has published the description of a dilator of the urethra, for the urethroscope. This instrument is composed of three metallic rods, all introduced together, and capable of being separated, with the ocular extremity surrounded with a funnel. The dilatation made, light was to be thrown through the funnel by means of a reflecting mirror. When a description of this instrument appeared, it had then not been tried, and I think that since it could have given no useful results. The inventor, moreover, only suggests its use for the anterior portion of the urethra, in front of the pubis. M. Hacken also proposed to examine the female bladder by means of tubes and a funnel, by which light might be transmitted as in the preceding case. Both of these experiments, I feel justified in saying, are unavailable.

Thirty years, at least, have passed since the efforts of M. Ségalas were made, and no ône, to my knowledge, thought further of the possibility of using vision in the study of urinary diseases, until, in the fall of 1852, I commenced to investigate them. Towards the end of the same year, I placed in the hands of the Imperial Academy of Medicine, a sealed paper, and on the 29th of November, 1853, I showed the instrument, about as it at present is used. I continued my attempts, and two years later, in a new communication, I gave the results of my first experiments. In 1855, the Imperial Academy of Medicine, appreciating the endoscope and what might be gained by it, rather than my services, already recognized, gave me the honor of dividing the Argenteuil prize, after experiments made before the committee.

The instrument was known, but it was necessary to prove its efficacy and adaptability. When, in its infancy, it was shown to some of my colleagues, one of whom remarked:—"We can see well with your instrument, but what is the use of seeing?" This was a question I was not prepared to answer, save by generalities, and so I thought it best to keep quiet for the time being. Now, however, after longer experience, I can say *what good sight does in the study and treatment of these diseases.*

The principle upon which the endoscope is constructed is very simple. A sound suited to the passage it traverses, to transmit the rays of light, a mirror pierced in its centre and placed obliquely at the face of the sound, for the purpose of throwing a faggot (so to speak) of luminous rays parallel to its axis—such are the essential parts of the endoscope. But, as this instrument is designed to aid the investigation of deep-seated organs, too narrow for the speculum to penetrate, it is necessary to throw as much light as possible, and at the same time guard the eye of the examiner from that light. Indeed, the instrument, for examination, and especially for operations, should be manageable to a high degree, and easily directed in every way.

For the purpose of increasing the light, a plano-convex lens should be placed between the light and mirror, so as to concentrate the rays of light upon the parts of the lower extremity of the sound or catheter. Opposite to the lens, there is a concave mirror, the centre of whose spherical surface coïncides with the lighting points, so as to reflect the rays of light received by it, according to their angle of incidence, and to cause them to strike the lens as if directly thrown upon it.

The means of lighting are of great importance. The luminous points near the focus of the lens are useful, the others are useless, if not injurious. Hence, it follows that we must have an intense light of small volume. Large flames will not do; candles, oil-lamps, and petroleum are useless; gazogene (a mixture of alcohol and turpentine) seems to me the best, its flame is intense but small. and the lamp well adapted to the instrument. Before I settled upon this lamp, I thought of the electric light, but it is too cumbersome to be carried around, and requires an assistant. It would, moreover, double the price of the instrument. Sunlight, so convenient for the laryngoscope, would not answer for the endoscope, because its rays cannot be controlled, and we must control light to make it useful in the employment of the endoscope.

There is yet another light that I believe may be good, but that I have never tried, it is the Drummond light. It might be useful in an amphitheatre, but would be difficult to use in private practice. Gazogene is, at present, according to my idea, the best.

[The author then goes on to describe the endoscope. To do this effectually, many terms, mechanical and physical, have to be used. Of some of these the dictionaries are often silent, others are so abstractly defined that none but a mechanician or student in natural science could understand them.]

(He says it is adapted to the study of the urethra and bladder; also of the neck of the uterus, but not of the internal surface of that organ, which cannot be explored without previous dilatation.

In the rectum, he says, this instrument lets us make profound, deep-seated examination, "so that I have been able to examine strictures beyond reach, and ulcerations also beyond the finger's touch."

He expects to publish, at some future time, the results of a pilous cyst of the ovary, with a fistulous opening, frequently examined by the endoscope. In wounds, this instrument, he says, may be very useful. Though a bullet may be recognized by the common sound, we may be called on to see it.)

Now let vs commence the study of the urethra:—

In the commencement of this subject, let me call your attention to the fact that the ENDOSCOPE does not act as a substitute for all other means of exploration, but only completes them, in adding touch to sight. Thus, before using the endoscope, we shonld use all other means of exploration, and especially of metallic sounds and of bougies with rounded ends. This examination will prove useful, not only as to our diagnosis, but will also direct us as to our use of the endoscope. Thus we will find out the sensibility of the canal, its deviations, and the obstacles which it presents; we can pursue the points upon which we should especially direct our attention.

If the canal is free and readily admits the sound of the endoscope up to the bladder, we should take advantage of it, and examine its whole length. If there is nothing to indicate to us the seat of the lesion, we may rest assured that we will find it, and if any sign, such as exalted sensibility, denotes a local affection, a complete examination will serve to show if any other point is affected.

An endoscopic examination should, as far as practicable, be made in a darkened room, as should all other examinations requiring artificial light; though a demi-obscurity will often answer, complete darkness is the best. As for position, the patient should be placed as if to be examined by the speculum. That position is well understood by all.

Before proceeding to the examination, preparation must be made for cleaning out the part examined. This is done by carded cotton so carried in as to wipe out all fluids calculated to obscure the view and keep it off the diseased structure.

All being ready and the patient properly placed, the sound is gently introduced as far as the vesical orifice. This we can judge of by the direction it has taken, by the depth it has penetrated, and also by the resistance it has to overcome before going farther. This resistance once perceived, we should be careful not to overcome it, for fear of going into the bladder and filling the sound with urine, and to empty it is an operation fatiguing alike to surgeon and patient.

It is unnecessary to say aught about the introduction of the sound. The introduction of the endoscope must first be based upon a knowledge of introducing this common and important instrument.

In moving the endoscopic sound forth and back, it will be found necessary to remove, by pledgets of lint or of cotton, such fluids as may obscure the view of the mucous membrane, be they mucosities, or only the oil used in greasing the instrument.

Whenever anything diseased is thought to be seen, it is necessary to clean out, and sometimes it may be well to pass the instrument near the bladder. By such movement, there is a change in the appearance of the mucosity under the lights, and certain details may be detected which would otherwise escape notice.

It sometimes happens that, in removing mucosities, the cotton, badly fixed, slips off. If the examination is over, withdraw the instrument and the cotton is thrown out at the first urinal emission; but if the examination is not over, if it is feared that the stream of urine may be too weak to throw it out, or if it is impregnated with a caustic which we do not wish to continue too long in the canal, it can be easily removed. In case of a preconceived obstacle, once reached, do not attempt to pass it. That is generally the point to study it, and the anterior portion of the canal can be investigated in withdrawing the sound.

To understand the diseased condition of the urethra, we must first understand its healthy appearance, and then we will commence.

The internal surface of the urethra, in its healthy condition, is smooth and polished; the endoscope shows neither the lacunæ of Morgagni, nor the orifices of the excretory ducts which cover its surface. This can be readily understood by the difficulty of finding them, even when the canal is largely opened; fortunately, it is not necessary to see them unless diseased. The same is true with reference to the ejaculatory canals. I know it has been claimed that they could be seen by means of the endoscope, but I have never done so, and I must think that an observer, in such cases, has been a victim to illusions which he might have avoided if he had kept more in mind the rules of examinations and dissections. The verumontanum is not visible; at least, I have never seen it, and I think its lack of consistence renders it liable to be obliterated by the sound.

Generally, authors describe the appearance of the urethra according to its appearance after death, thus, according to them, its color varies in its different portions. These different colorations are all of cadaveric origin. The blood, obeying its weight, collects in the most dependent parts, and soon, by imbibition, stains parts less elevated, whilst all others, more elevated, remain uninjected.

M. Sappey understands this; by removing the blood from the veins, by means of water injections, he has, in a manner, proved that the mucous membrane is without color throughout its whole course, MM. Rickert and Jarjavay describe it as slightly yellow. Such is, undoubtedly, its color during life; only this yellowish tinge, which is the foundation of the coloration, is more or less intense in different persons. It thus takes on very much the complexion tint of the skin, a circumstance often mentioned by those to whom I have shown the healthy urethra. This tint is uniform from near the meatus, where it is more red.

It is generally believed that the mucous membrane of the urethra forms longitudinal pleats. This opinion is the result of opening the canal lengthwise, or else by dividing it transversely by sections. This last is certainly the truest method, as the first-mentioned permits the mucous membrane to pleat in any but a natural way. Transversal pleats have also been described: M. Sappey denies their existence, and attributes their discovery to a desire to make a name. I must say that such reasons have often been used to excuse the difficulty of penetrating to the bladder with a small-pointed sound, and that too though a healthy condition of the canal existed—these pleats may be produced by the improper introduction of this sound. I must confess to have sometimes, formerly, encountered them, but I attribute them to the before-mentioned cause. The endoscope has never shown me anything like them. As to the longitudinal pleats, they are not very marked, but they can be seen by the endoscope. In withdrawing the instrument and following the axis of the canal, the mucous membrane can be seen to contract upon the end of the sound, but when these pleats form after the urethra has lost its elasticity, they are at a certain distance from the instrument. Thus the walls of the urethra, in closing, form a funnel, the summit of which is truly pleated like a (*cul de poule*) pullet's tail.

These pleats are not so regular as anatomists generally state them to be; their number and disposition is irregular, they vary according to the direction of the sound, and by pressing the sound upon different points of the urethral circumference, this linear contraction can be caused in the part pressed. It is necessary to be familiar with the manner in which the urethra closes over the sound, for if it does not do so uniformly and regularly, we must believe that there is a pathological alteration, by which the suppleness of the walls of the urethra is impaired.

Now that we know the normal condition of the urethral mucous membrane, as shown by the endoscope, we can investigate the diseases of the same membrane. Let us commence by acute urethritis, a disease which plays an important part in urethral pathology, and which may, in some means, be considered as the father of many complaints.

Writers have frequently confounded urethritis with blenorrhagia, this, I think, is a great error. Urethritis is a disease, an inflammation, produced by many causes. Blenorrhagia is a disease produced by a special or specific cause, and transmissible contagiously, always preserving its peculiar characteristic of transmitting effect to cause, or, if you wish, cause to effect.

Blennorrhagia being more frequent than all other urethrites, may possibly be the cause of all the confusion existing in this diagnosis. Now we must state that, according to the symptoms alone, the difference is slight, and only found out in the march of the disease, and its cause, when chronic and when the lesions are consecutive. So long as the acute stage continues, inflammation dominates and masks the peculiar characteristics of each affection.

In this respect, urethritis is like other inflammations; similar examples exist in all the organs. Suppose, in a case of orchitis, seen for the first time, you wish to distinguish, at once, the class to which it belongs, many times you cannot tell whether it is due to a contusion, a blennorrhagia, to epidemy—or mumps and consequent inflammation of the testicle, or if it is the commencement of a tuberculous orchitis; if closer and more decided examination of causes, and of other organs, does not enlighten the diagnosis, it will be necessary to abide your time, until the progress of the disease may enlighten you. Is another example needed, the pathology of arthritic blennorrhagia differs but little from orchitis, and it is not difficult to find cases in which we cannot tell the origin of the disease. When a knee is painfully inflamed, with acute or sub-acute effusion, attempt, throwing aside causes, relying only on the local symptoms, to decide if the arthritis is rheumatic, traumatic, or blennorrhagic; this you can but rarely do, and oftentimes the march of the disease will not enlighten you. I recollect a case at the Cochin Hospital, embarassed with such symptoms as to render it almost impossible to distinguish the kind of arthritis I was treating; the inflammation was not very acute, there was but little fever, and, though the knee was the seat of a painful effusion, the patient, according to his say-so, had only felt a sensation of weariness, the result of overwork, by which to explain his feverishness. The local symptoms consisted in a hydarthrus, with considerable pain, caused either by motion or pressure; at first, the skin a little warm, but not red. Now for the cause: the patient had an old blennorrhagia, had frequently felt rheumatic pains, and, when attacked as I mention, was engaged in weeding in damp ground, the affected knee being down. I must confess that the patient was cured without my ability to diagnose whether the arthritis was blennorrhagic, rheumatismal, or traumatic.

These examples are sufficient to show that in relying alone, as has been proposed, upon a concurrence of the symptoms, for the purpose of establishing the nature of urethritis, we may be deceived, as has happened, and confound under the same name traumatic, hepatic, catarrhal, blenorrhagic, and other discharges. If the term blennorrhagia is to be employed as the designation of all urethral discharges, then I cannot tell how to term that species of urethritis which is the common, the most important, and the best characterized, that which is generally understood by blennorrhagia. We will acknowledge inflammation of the mucous membrane of the urethra can be caused by whatever may give rise to inflammation of other mucous surfaces, but at present our attention will be especially directed to blennorrhagia, the most important, and we will only touch upon the others, for the purpose of distinguishing. It is not intended to give here a complete history of this disease, but to seek what lights the endoscope can add to those already possessed by science.

What I just said of the different inflammations of the urethra will find its proof in what is to follow on the same subject, but, for the present, we must admit, in anticipation, that by blenorrhagia, we understand a disease always due to contagion, and which, in its complete development, if it does not cure spontaneously, or by proper treatment, passes to a chronic stage, and finally terminates in stricture of the urethra, which is its last stage, as a cicatrix is the last stage of ulceration.

Some days after contamination, the patient feels a burning towards the meatus, then itching, and then pain, which rapidly increases. The lips, too, of the meatus are glued together by thick mucus. The pain soon extends up the urethra, the entire length of which it invades in a few days; and it can even reach the neck of the bladder, where it produces symptoms which we are not here called upon to study. We only mention the tendency of the inflammation, commencing at the meatus, to extend to the superior parts of the canal. In proportion as the disease extends, the discharge increases, becoming yellowish or greenish, according to the intensity of the inflammation, to again assume the white color of muco-pus as the pain diminishes. Endoscopic observations have enabled me to follow the march of the disease, and to recognize by the sight (*de visu*) the characters of the inflammation. During the first few days, whilst the disease is in its most acute stage, the examination is impossible, on account of the intense pain caused by the introduction of the sound. The most recent case that I have examined was of eight days standing, and it is rarely we can explore the urethra so soon as that. At that period, the inflammation extended to the anterior half of the canal; through this extent, the mucous membrane was of an intense redness, it is without polish, and presents the appearance of the superficial which form upon the glans in balanitis; in other words, it had the appearance of a mucous membrane inflamed and derived of its epithelium. In more advanced cases, the lesion does not change its character, but extends nearer the posterior portion of the canal. It finished, in this way, by successively reaching the bulb, the membranous portion, and the prostatic portion, which I have only found diseased after six weeks or two months duration. At this period, an important phenomenon takes place, one which all of the exterior symptoms would not lead us to anticipate. The anterior part of the urethra reässumes its healthy condition. In some cases, indeed, whilst the inflammation possessed all of its peculiar characters in the bulb and membranous region, the anterior portion of the canal presents a polished surface and natural color. The prostatic portion, in which this disease may also locate itself, was also cured in many cases, for it has frequently happened that I have found the characteristic lesion confined to the bulb, whilst the anterior and posterior portions were healthy, in cases which had presented all the signs of an extension of the disease to the prostate and neck of the bladder. I have several times had occasion to exhibit analogous cases in this amphitheatre.

Reduced to a limited extent, the inflammation seems to become deeper seated, the redness of the mucous membrane is more pronounced, its surface more uneven, its surface is not simply unpolished, it presents salient points more and more numerous, and tends, too, to take on the characters of the lesion that we will soon study in speaking of chronic blennorrhœa and of commencing strictures. In a word, at this period of its march, blennorrhagia is upon the point of passing into the chronic state, if it has not already reached it. We will follow its ulterior evolutions in our next meeting.

But I must, at once, cause you to observe that it is difficult to admit, as has been attempted to be established, that blennorrhagia propagates itself by an inoculation, step by step, little by little, of the secreted matter, since we see parts retake their healthy status and not again become diseased, though constantly bathed by the muco-pus coming from uncured portions.

We can conclude, from these observations, that those authors who, like M. Cruveilhier, state that the discharge comes from ulceration, are entirely right, as the mucous membrane presents all of the characters of superficial ulceration. But let us pass to the study of the lesions which may be confounded with the one we have just described, and let us commence by recalling the fact that in acute inflammation the local mucous lesion varies little or none, according to circumstances, and the distinctive local characters scarcely commence to show themselves until the malady passes into the chronic stage.

Acute urethral inflammations, not blennorrhagic, are due to causes traumatic, herpetic, or catarrhal. Traumatic urethritis is almost always caused by solid foreign substances introduced into the canal, either accidentally or with a surgical intention, such as sounds or bougies, or by fluids, as injections. Such cases of inflammation are rarely very acute; often the endoscope shows only a simple redness of the mucous lining, which otherwise retains its natural smooth condition; if greater acuteness occurs, the epithelium becomes detached and the red surface is at the same time roughened, as in blennorrhagia. The tendency of the disease is towards cure as soon as the cause is removed, it does not migrate towards the interior, as we just described, and becomes extinct through its whole extent at the same time, without contracting upon one point of the urethra. It must be understood that we are now speaking of simple traumatic urethritis, and not of that which, finding in the membrane alterations consecutive to blennorrhagia, excites their reäppearance and leaves them to continue after they (the traumatic) are removed. This subject will soon be rendered clear by the study of the endoscope in blennorrhæa.

Herpetic affections can, undoubtedly, cause acute urethritis.

Acute herpes, especially in children and men who have not had sexual intercourse, is one of the most common causes of balano-posthitis, and discharges are frequently met with which it seems impossible to refer to aught else than herpetic patches in the canal. But, up to the present, I have not directly observed them, at least in acute discharges, though they may be seen in the chronic flow. I will only remark that, in such a case, the affection presents all the mobility of acute herpetic diseases, and there is such difference in the march and irregularity in the seat, as sometimes to guide in the diagnosis.

Catarrhal urethritis seems to be rare, as I have already stated. I have only met with two cases in the acute state, or, if I have seen it in other cases, twice I have observed it under such circumstances that I was perfectly sure contagion had nothing to do with its etiology. In my two cases, it seemed like the last phase of an acute general catarrhal affection; in one of them, then my companion and now my confrère, the disease commenced as a coryza, then extended to the bronchia, then to the digestive organs; at last, after more than three weeks, just at the moment when, the intestines throwing it off, the patient thought himself cured, the fever greatly increased, accompanied by pain in the kidneys, then a vesical catarrh, and, at last, by an acute urethritis, which, in its turn, disappeared, having resisted both copaiba and cubebs. In this case, and in the other, which it is unnecessary to relate, the urethral pain, starting from the neck of the bladder, extended through the whole length of the canal, following a route inverse to that demonstrated as usual in blennorrhagia. This march, I think, may be considered as constant in urethral catarrh, and can serve as a diagnostic base.

Though, in acute urethritis, with which we have just been occupied, the endoscope does not play so important a part as in the chronic affections which will be the subject of the ensuing lectures, I believe, however, that we owe to it the ability to establish a marked separation between affections generally confounded, and of the diversity of which, I think, you can no longer be doubtful. This distinction will soon be more clearly drawn, and will enable us to comprehend how an affection, hitherto regarded as unique, in spite of the absence of sufficient signs, is sometimes contagious and sometimes not, disappears in one case not to reäppear, and in another returns without apparent cause, and continues indefinitely without giving rise to grave lesions, and at other times terminates in stricture and its consequences.

### SECOND LECTURE.

### *Blennorrhœa.—Granular Urethritis.*

GENTLEMEN:—In our last lecture, we studied the lesions produced by blennorrhagia, from its inception to the moment when it loses its acuteness. We will now proceed to investigate the chronic discharges from the urethra, and, as we reserved the name blennorrhagia for the contagious affection for which it was created, so we will call blennorrhœa the chronic discharge, only, which springs from the same cause. and we will endeavor to distinguish from it especially the herpetic discharges, which have often been confounded with them, and which, by their lightness, should be to us only a secondary consideration.

Discharges of a blennorrhagic character, though often so slight as to attract but little attention, are the cause of almost all strictures, by a mechanism, the study of which we can make by the endoscope. If authors have not insisted more upon this fact, and if some have not even perceived it, it is not only because blennorrhagia generally presents symptoms too slight to attract attention, but it is also because discharges from other causes, and especially herpetic, confounded with them but without the same consequences, obscured the question and rendered it difficult to perceive the connections. And this confusion, we must acknowledge, was difficult to avoid, for, without endoscopic aid, the symptomatic differences are almost null, and certainly not sufficient to establish a diagnosis; so nothing was left but the march of the disease and the concomitants give us any clue to its nature.

We left blennorrhagia when it was confined to the deep-seated urethra, when it was fixed upon the bulbous, membranous, and prostatic portions, when, at the same time, its acute symptoms were gone. According as it may persist in either of these regions, its sequences differ; so we will, at present, investigate the evils attendant upon the bulbous and membranous regions, reserving until later those peculiar to the prostate.

Blennorrhagic symptoms, when once weakened, become more and more so, so as ultimately to be scarcely recognizable or perceptible. The pain becomes more disagreeable than truly painful, so much so that patients often compare it to a disagreeable heat or burning, to a weary tickling, deep seated in the perineum, to points corresponding to the bulbo-membranous portion of the urethra. This sensation is rarely continuous; it generally occurs when the urine is about to be evacuated, and continues for some time afterwards. Often it is perceived before and after but not during micturition. Some patients do not feel it at all; but most frequently when they say it is not felt it is for want of attention, which, once called to the fact, they perceive it, as do others.

The discharge follows, in its decrease, the same march as does the pain; in some lymphatic temperaments, there are a few drops of a turbid, whitish, slightly opaque liquid, which may spontaneously escape from the meatus and not soil the linen; but generally this matter is so scarce that, without exacerbation, it is completely cleaned out by the urine. Sometimes it can be recognized in the form of filaments thrown out by the first jet of urine and found swimming in the urinal. If we wish to be assured of the existence of this discharge, it must be sought for when the patient has not for some time urinated, and especially in the morning on awaking; then, in pressing from behind forwards it is rare that we do not bring out a drop of tenacious fluid, limpid, or slightly turbid, small, usually, but visible between the lips of the meatus, which we also find generally glued together by a dry mucus, be it during the day or only in the morning; or there may exist no trace of discharge, other than viscous filaments, which extend between the lips of the meatus when they are separated.

A last sign experienced by the blennorrhagic patient, is difficulty in emitting urine. This difficulty, often very great in the acute stage, diminishes and finally disappears, so that the only trouble which remains for some time is the bifurcation and gimlet twist of the urinary jet. These two phenomena, which only occur accidentally in the healthy state, are due to the viscous mucus which sticks to the walls of the urethra and resists the first impulsion of urine, but this is only temporary; after an uncertain and unfixed time, the urine is thrown not so far, the jet is smaller, the bladder is more slowly emptied, the patient experiences a dysuria, which, if left to itself, goes on increasing.

To the acute inflammatory swelling which caused the first stricture, a chronic lesion succeeds, which favors a swelling slow in its march, but continuous in its progress, and which will, sooner or later, produce stricture with all of its consequences. Hence, we can foresee that, in every blennorrhœa, there will be stricture. Indeed, such is the case, whenever the lesion is not confined to the prostate; but we must expect to find a stricture, as it is generally understood, from the commencement. A stricture, such as will arrest sounds and bougies, is only found at a more advanced period. When blennorrhagia is near its commencement, the swelling is slight, there is, possibly, only a rigidity of the inflamed surface instead of its natural suppleness and rigidity; it is necessary, for the purpose of finding an obstacle, to use a bougie of proper size, with a bullet end, and to use it dexterously and carefully; then, in advancing upon, or withrawing towards, from behind the lesion, a gentle resistance is felt, and the patient complains of a slight pain which he does not experience, save when the bullet end of the bougie is attacking the stricture.

The bullet bougie also is capable of aiding us in our diagnosis of urethral ulceration; it is rare that, when it encounters the characteristic ulceration of blennorrhœa, it does not show more or less of a bloody tinge, though no violence, sufficient to wound the membrane, has been used.

If we now establish a comparison between blennorrhœa and herpetic discharges, we will not find, in the symptoms just related, sufficient wherewith to establish a positive diagnosis.

The discharge is not different; the pain is more itching than otherwise, but this characteristic, though often well appreciated by the patient, is too slightly marked for us to attach much importance to it.

The urinary jet is not diminished by the herpetic plates, and the bullet bougie does not give evidence of their existence: the first of these signs is absent in commencing blenorrhœa, and the second is so slightly marked that its absence should not affect the diagnosis. In fine, these symptoms, interesting in the study of the disease, cannot enable us to diagnose between the different species of discharge, if the endoscope did not aid us in the discovery of the blennorrhœa.

In our last lesson, we saw that blennorrhagia, in its chronic state, fixes itself in the bulbo-membranous portion of the urethra, and that the mucous membrane of the diseased part, at first simply unpolished, soon becomes rough and uneven; these inequalities increase, multiply, and, in the end, become more prominent, and granulations are formed. Then the diseased portion presents a surface dark red, uneven, sown with rounded granulations, sometimes separated far apart, and then at other times so nearly together as to cover the entire diseased surface. The membrane, at this point, presents the appearance of a mulberry, according to the spontaneous remark of one to whom I showed the lesion, as well by reason of its color, as on account of its granular aspect. The granulations vary in size from a grain of mustard seed, to that of a grain of millet, or even still larger; the smallest seem to be of more recent origin.

In the cases we are now speaking of, the lesion confines itself to the bulbous and membranous portions of the canal. They constitute what we may call granular urethritis, which shows some resemblance to granular metritis, and still more to granular conjunctivitis, owing, doubtless, to the fact that, in anatomical disposition and vital condition, the urethral membrane more nearly resembles the conjunctiva than the membrane of the uterine neck. It is impossible, when we examine these three affections, not to perceive a similar lesion in all, the expression of a like malady in three different organs. The granulations are almost always of a dark red color, often like wine settlings; but sometimes I have found in their midst other granulations, less numerous, small, and of apparently a greyish color, such as might bring it still nearer to the conjunctival granulations, as described by M. Wecker. We will return to this similitude.

These granulations may occupy more or less of the length of the canal, most frequently from a quarter to half an inch, sometimes the whole posterior part, from the termination of the spongy portion to the vesical orifice; but there is one characteristic almost ever present, it is a unique lesion, be it ever so much or so little extended, there is no interruption between its two extremities, there are no isolated places, separated by healthy surface. The granular ulceration is only found in a single point, more or less extensive; before and behind the diseased portion there is a redness that diminishes as it recedes from the seat of the granulations.

The herpetic ulceration is very different, and, with some attention, it can be readily recognized. But, before entering upon this subject, gentlemen, I ought to advise you, that if I use the word herpetic, it is only because this species of ulceration corresponds exactly with the herpetic plates that we observe upon the skin, upon the lips, and the neck of the uterus, and not because I regard them as products of the dartrous affection. On the contrary, I think that many of these ulcerations are of an arthritic nature, according to the circumstances under which they are produced, and the characters of the external eruptions with which they are often allied. I adopt in this, the ideas of M. Bazin upon gouty affections.

Not yet having the local signs which will enable me to distinguish between arthritic and herpetic ulcerations, I confound them together under the most popular denomination. Generally, the herpetic ulcerations are multiple, they are found in different parts of the canal; besides, they have a fleeting character, such as are seen in aphthous affections of the mouth or on the uterine neck. The patch which we have found in our examination is not often re-found, but others are found in different places. These ulcerations differ from the granular species, by being generally of much less extent. We must add that the characteristics are different; their surface is not granular, often it is only unpolished, as in the case of aphtha attacking the inside of the jaws, or in those cases where plates of epithelium are removed from the mucous membrane, as is frequently the case in the buccal membrane with those who smoke much. They approximate, then, blennorrhagic ulcerations of recent date, but the characteristics which I have just given you are distinguishing, and, moreover, we cannot confound them, if we remember that in blennorrhagia the ulceration appears to its best advantage when the inflammation is still at its most acute stage.

There is still another form of herpetic urethritis, which seems to be more deeply seated; of this I have just shown you a case. The ulcerations presented by this affection vary less, as to their seat, than the preceding; they are of uneven surface, and, without attending circumstances, a superficial examination would induce us to call them blennorrhagic ulcerations, just forming; but a more considerate examination shows us that, instead of prominences, the irregularities are caused by depressions. Whilst the granular surface may be compared to a mulberry, it is more like the depressions which we find in an orange skin, or in a tailor's thimble. Before going further, permit me to compare these affections of the urethra with similar affections of the uterine neck. In this last organ, granular ulceration is the same as in the urethra. Since I commenced studying the distinctive characters of this affection, about five years ago, at the Loucaine Hospital, I have never seen, upon the same subject, but one kind of granular ulceration, commencing in the lips, or else in the cavity of the neck, and extending more or less, but never separated by healthy surfaces.

The herpetic plates are most frequently multiple, and irregularly scattered upon the surface of the neck, and often show depressions and small cavities, such as we have just described, and such as we have compared to those on a thimble. When we examine, on the uterine neck, very recent herpetic eruptions, we frequently find small vesicles grouped; but if on the morrow an examination is made, the vesicles are not seen, but, in their stead, gutters, (*capules*,) the formation of which is, to my understanding, very comprehensible. Though I have never seen these vesicles in the urethra, I think these same gutters (*capules*) should form there by the same mechanical influence; but they must be less persistent, on account of the different anatomical formation of the two different organs, and, consequently, less easily found. But they constitute, no less, a decided point in differential diagnosis. The distinction which we just established amongst the different characters of discharge, often confounded, and which the employment of the endoscope enables us to refer to lesions of very different natures, will enable us to comprehend the peculiarities which have been observed in the march of blennorrhœa, as it has generally been understood. Blennorrhœa, or granular urethritis, synonyms, according to my view, are partially chronic in their nature and tend decidedly towards stricture.

The pain, or rather the tenderness, which replaces it, augments or decreases with time only; the same is true with refer- to the discharge, but the trouble in discharging the urine goes on increasing, and dysuria, at first slight, insensibly increases to an extreme degree, until stricture is entire and complete. The course of this disease is affected and influenced by but few extraneous circumstances. Atmospheric influences and changes of the seasons are without visible effect; improper diet and fatigue are alike inert to produce an increase of pain or discharge, but sexual intercourse will increase the violence of the symptoms, and often re-bring, for some days, a sub-acute inflammation. When the affection is of long standing, and a stricture, already formed, is surrounded by an inflammatory engorgement, too much driving, horseback, or carriage exercise may increase the dysuria. The same is true, with reference to an excess at table at any time, but a few days puts all right, and all goes on in its uniform order.

We see, also, discharges which completely disappear and then reäppear, with all of the accompanying pains, and know no reason for it; we see them, not very rarely, break out once or twice a year, sometimes more frequently, but always about the same epoch, most frequently in the spring, and then in the autumn; and then, if I follow my own experience, winter succeeds, third in order—the hot season is least of others prolific in such cases. I have met with cases recurring every spring, for several years, forming what is called repeated blennorrhagia (blennorrhagia à répétition); others, I have seen, after a considerable lapse of time, and especially after a decided change of weather, to harsh, cold, or damp. In similar cases, the endoscope will show in some part of the urethra, be it above or below, frequently in many points, herpetic patches such as I have described, but it will not show granulations. The season most favorable to the production of rheumatismal affections is most favorable to the production of this, thus I will call your attention to the propriety of connecting these affections with those of the tegumentary system, classed by M. Bazin as arthritic (*arthritides*).

Does true blennorrhœa ever reäppear? Decidedly not, provided the cure has been complete; but if granulations remain after the external symptoms have almost entirely disappeared, we often find a variety of repeated blennorrhagia (blennorrhagia à répétition.)

For a long time, I saw a patient who had left off treatment before the cure of the granulations. According to his own expression, there was a slight humidity of the urethral canal, and the emission of urine was slightly disagreeable, but so little as not to cause any especial annoyance. Each time, however, that he had concupiscent connection, he asked of me the aid of the endoscope, which always showed granulations as large as ever. Traveling, drives, and horseback exercise produced no effect, but, invariably, sexual connection brought on a relapse. Similar cases are not very rare, you have all seen men who cannot employ their genital functions without becoming subject to a discharge; whenever I have examined such, I have inevitably found urethral granulations.

As I have already said, the march of blennorrhœa is essentially chronic; we will follow it in our next lecture, up to the moment of its disappearance, after having produced stricture; but without proceeding so far it may last months, and even years, without losing its granular character. I will here mention the particulars of a case in which the granulations continued for above eleven (11) years. I have seen cases of longer date, but the following, the first of the kind subjected to an endoscopic examination, furnishes a complete example of the affection:—

D. came to me in October, 1855, with an acute orchitis. He assured me that for a long time he had not had blennorrhagia, but a slight and clear discharge remained as a sequence of an acute blennorrhagia contracted in 1846. Generally he paid no attention to it, but whenever he squeezed his urethra before urinating in the morning, some drops of clear but stringy fluid were discharged. I soon found out that his urinary jet was very small, twisted, and fell almost vertically. The epididymitis once cured, the patient, somewhat embarassed by dysuria, submitted to a full examination. In the bulbous portion of the urethra, I found, by aid of a bougie, a stricture, the passage of which was painful and blood came back on the bougie, the retracted part of the urethra was about four lines in extent. I applied the endoscope, and found, at the point of the stricture, an inflamed surface, red, and covered with granulations of the size of a grain of millet, some larger, some smaller. In a word, the case was similar to an affection of the uterine neck. In withdrawing the sound, the granulations disappeared and a temporary redness only remained; all other parts of the urethra were healthy to the meatus.

Once this lesion found out, it strikes me that the nature of the disease is explained; it is easy to comprehend the persistence of a discharge kept up by granulations similar to those often found in the conjunctiva and uterine neck. This ulceration also accounts for the chronic subjacent engorgement which produces the stricture. These granulations, so often found, notwithstanding what many authors say, these persisting discharges often complete dilatation of the strictures to which they were attributed, and which reäppear as soon as the use of bougies is stopped, are the cause of this trouble.

Although I concluded to treat this lesion of the urethra as I would a similar one in the uterine neck, and following from cause to effect, as existing between ulceration and stricture, I concluded to use no direct means against the last named.

The endoscope, moreover, enabled me to act upon the diseased part, as we do by means of the speculum upon a diseased uterine neck. A concentrated solution of nitrate of silver, placed upon the diseased points by means of a canula, and the effects of which I could verify by sight, sufficed. These cauterizations were repeated every three or four days. During the interval, the patient used an injection of the decoction of roses (*de peonies*). Though the injection was one part of silver to three of water, the pain was moderate.

This treatment, commenced the 5th December, 1855, was continued more than a month, when, on the 25th January, 1856, I passed the sound into the bladder. It passed the stricture without my perceiving any obstacle, and filled, entirely, the whole anterior portion of the urethra. Thus, it was evident that the stricture had disappeared, simply because the granulations had. I could then examine the whole of the urethral mucous membrane, from end to end. The prostatic region was slightly red; this redness was never decided in the membranous portion, and towards its union with the bulbous; granulations, somewhat ulcerated, were found, but scarcely visible. Moreover, the patient assured me that he urinated as well as when young. These cicatrizations were sufficient to abolish all trace of these granulations; then he continued the treatment for two days and then stopped. A few days afterwards, the discharge ceased entirely, and the bougie, upon its introduction, met with no obstacle. The endoscope showed only a slight redness in the affected part, and at the same time the stricture had disappeared without any special treatment. This case I have watched for eight years, the cure is complete.

I have described to you, gentlemen, the normal form and appearance of the urethral granulations; but they do not always present the same characters, as you will see in the future. Really, in many of the granular affections of the urethra, the granulations swell, lose their hemispheric form, become softer, and take on the appearance of fleshy granulations. Then, in the field of the instrument, we find a surface similar to that of a suppurating sore. The movement of the sound will enable us to judge of the consistence of these conical formations (*bourgeons*). When this transformation takes place, the discharge becomes more abundant and more purulent, so much so as to stain the linen. It seems that the erosion is deeper seated, even ulcerated, at the same time it much more readily bleeds. This alteration may be much more complicated: the depressions may become larger, softer, more unequal, nearer together, and of a dark red color (*lie de vin*), the ulceration takes on a fungous form, bleeding at the slightest touch, and of sweating blood, as it were, so as to conceal the other parts; wiped off, it almost immediately reäppears, scarcely giving time to see the ulcerated surface. It is not even rare to see these fungosities bleed so readily, that the gentle passage of a bougie may cause the discharge of blood from the meatus. This same form of granulation is found in the conjunctiva, but it is especially and frequently found in cases of granular metritis. Thus, we find this same granular affection in the same organs most frequently attacked. When, by means of caustic applications, the vegetating character of the surface is lessened, the granulations retake their ordinary appearance and furnish evidence of the true character of the disease.

We have seen that these granulations last long; left to themselves, they cure, leaving after them a lesion which remains as long as a cicatrix after a wound. Let us now proceed to study their termination. They cannot exist without a sub-acute inflammation in the mucous and sub-mucous tissues; hence, the swelling of these parts, and, consequently, the stricture, a stricture at first scarcely observable, but which, gradually increasing, ends by becoming a decided obstacle to the emission of urine. During an uncertain period, this swelling, increased by the infiltration of lymph, not yet organized, forms of itself the stricture. If, at this time, the granular ulceration is cured, the ulceration disappears and, consequently, the infiltration and stricture are relieved. The length of time during which the tissues may retake their normal condition is, sometimes great. You may remember a case I mentioned to you, in which the coarctation yielded upon the cure of the granulations, after a continuance of more than eleven years. However, so long as this condition lasts, during which the infiltrated fluids are susceptible of reäbsorption, it is impossible that they remain indefinitely in the tissues without producing changes analogous to those which are produced in external parts which may be the seat of chronic irritation, such as indurations or callosities. Besides, the inflammatory action will have reached the fibrous tissues which partly compose the urethra. Now we know that the fibrous tissue contracts under the influence of slow inflammation; thus, here is another cause of stricture, as demonstrated by M. Guérin.

When blennorrhœa has reached this point, the endoscope still shows the existence of granulations, but to cure them will not suffice to relieve the coarctation; the granular ulceration no longer controls the morbid effects which it has caused, and these effects continue to progress, independent of their exciting cause. In addition, without proper treatment, the granulations will not yet disappear. The infiltrated fluid becomes slowly organized, new fibrous elements are developed, as also elastic fibres, which, added to the fibrous tissue already retracted by the existing inflammation, causes the formation of a tissue similar to that of a cicatrix, or, rather, a true inodular tissue which takes the place of the healthy one.

In this transformation, the vascular element considerably lessens, so that the mucous membrane in that part loses its inflammatory redness and becomes paler than natural. But this is not the only change. The granulations disappear from absorption, ulceration, or because of the atrophy of the vessels which nourished them. As they disappear, the membrane, whose structure they have destroyed, takes on the appearance of a cicatrix, similar to a subjacent layers. Then the blennorrhœa is ended, the granular urethritis has terminated, and inodular strictnre takes its place. It is useless to press upon your attention the parallel which exists between these granular affections of the urethra and those of the conjunctiva. Keeping in view the different functions of these two organs, we readily see that the march of the disease, its effects, and its termination are essentially the same in the two cases. It is impossible to state the time necessary to heal the affected parts.

Sometimes, a year or more may pass before the infiltrated material proves itself perfectly organized; again, several years may pass before the disorganization of the affection can reproduce disease in which the termination shows a decided change. We have already mentioned a remarkable case, in which granulations continued for over eleven years, which yielded at length to proper treatment. But, on the other hand, we have seen cases with a remarkable slowness in adapting themselves to their cause.

A lieutenant in the French army, contracted a blennorrhagia in Bohemia, when this province was in the possession of France, say in 1813. When he consulted me, in 1860, he had a retention of urine, which had increased for some years. Moreover, since his first clap he had no other, but he had always had a slight discharge, to which he had paid no attention. His stricture yielded readily to the passage of bougies; but from day to day all that was gained was lost. I then supposed that the endoscope would be necessary for a correct examination and that ulceration existed. I really found the membrane very red, and many granulations, without the characteristic signs of stricture. In the treatment of the granular affection, attention must, of necessity, be paid to the ulceration; but for the purpose of conquering the stricture, dilatation must be resorted to. After the cure of these granulations, there is no rapid return, and thence patients are satisfied in passing, at long intervals, a large-sized bougie, which rarely meets an obstacle. Sometimes this granular urethritis may run on for forty years, without reaching its period of stricture, except at its second period, or stricture, where the former effusion is dependent upon a pernicious affection. I mention this fact as the longest limit of the disease, as reported by M. Ricord.

### THIRD LECTURE.

### *Complications of Blennorrhœa.—Strictures.*

GENTLEMEN:—We have followed blennorrhœa from its commencement up to its disappearance and when stricture succeeds it. Before examining and describing this disease, let us give some time to those cases which may complicate the preceding periods. The peculiar complications of blennorrhagia are cystitis, ophthalmia, arthritis, and orchitis. I shall spend but little time upon the first three named affections.

Cystitis is a frequent complication, and it is well known that it does not rarely occur during a case of blennorrhagia, and especially when this disease takes on a chronic form; but its connection with blennorrhœa has not been so decidedly marked. It sometimes happens, however, that the inflammation which accompanies urethral granulations, extends, by continuity, to the mucous membrane of the bladder. Many times you have seen patients, treated for granulations, attacked with cystitis. There are more than one case of cystitis that I could mention to you, which has been treated, after longer or shorter intervals, in various methods, without good effect, until the endoscope enabled us to see granulations in the bulbous or prostatic portion of the urethra. Often it occurs that the cystitis does not continue after the granulations have been removed, but in such cases the disease should not have continued very long, for the vesical inflammation would be too deep seated to cure itself. Many of the so-called vesical catarrhs are owing to the same cause, and are only called so because the blennorrhœa producing them has been overlooked. The herpetic and arthritic diseases may also give rise to cystitis, such as we have spoken of, and which reäppear from time to time; a careful study of the circumstances attending their development may afford us presumptive evidence of their character, but a very correct diagnosis can only be made by means of what the endoscope will show.

I have mentioned to you a casc of arthritis of the knee, the cause of which was veiled in doubt; this is not often the case; the existence of arthritis, when blennorrhagia takes on a chronic form is well known; granular urethritis, once well set up, may produce arthritis, the cause of which will be difficult to discover unless the discharge, not always easy to be found, is suspected.

There is another affection, intimately-connected with urethral granulations, I mean conjunctival granulations, or, to express it better, these two diseases are only one, attacking, at the same time, different organs. We have seen that the characters of the lesions are the same, and frequently, when showing to ophthalmologists the disease, by aid of the endoscope, they have recognized it as similar to the disease they were accustomed to study and treat on the eyelids. There are at present, in Ward St. Peter, several cases of granular ophthalmia, so that you can see for yourselves that the appearance of the conjunctiva, thus affected, is precisely similar to the urethral surface, subject to blennorrhœa. Appearance, however, is not all that identifies them; these two different maladies will each produce the other. It is a well-known fact, that the blennorrhagic urethral discharge will produce a blennorrhagic ophthalmia; this ophthalmia, if prolonged, becomes granular, as in the urethra. I have several times been enabled to observe this.

Amongst others, was a woman, treated in the special dispensary, for blennorrhagic ophthalmia. The treatment applied at each consultation, but remitted in the interval, certainly much relieved the disease without curing it, and it commenced to take on a more chronic condition, when she was admitted into my wards at the Cochin hospital, with double pannus and corneal ulceration. A continued and energetic treatment brought about a cure, but conjunctival granulations were observable.

The contagion is not confined to the acute stage, it can take place during its chronicity, as Professor Thiregtias amply and ably demonstrated. He has reported cases of conjunctival ulceration, produced by accidental inoculation from urethral granular inflammation, and he has also produced granulations in the uterine neck and in the urethra by thus inoculating the secretion from the conjunctiva. I have already drawn your attention to the identity of these two affections; the same is true

